# Development of a Quantitative Methylation-Specific Polymerase Chain Reaction Method for Monitoring Beta Cell Death in Type 1 Diabetes

**DOI:** 10.1371/journal.pone.0047942

**Published:** 2012-10-29

**Authors:** Mohamed I. Husseiny, Akio Kuroda, Alexander N. Kaye, Indu Nair, Fouad Kandeel, Kevin Ferreri

**Affiliations:** 1 Department of Diabetes and Metabolic Diseases Research, Beckman Research Institute of City of Hope, Duarte, California, United States of America; 2 Diabetes Therapeutics and Research Center, The University of Tokushima, Tokushima, Japan; La Jolla Institute for Allergy and Immunology, United States of America

## Abstract

DNA methylation is a mechanism by which cells control gene expression, and cell-specific genes often exhibit unique patterns of DNA methylation. We previously reported that the mouse insulin-2 gene (*Ins2*) promoter has three potential methylation (CpG) sites, all of which are unmethylated in insulin-producing cells but methylated in other tissues. In this study we examined *Ins2* exon 2 and found a similar tissue-specific methylation pattern. These methylation patterns can differentiate between DNA from insulin-producing beta cells and other tissues. We hypothesized that damaged beta cells release their DNA into circulation at the onset of type 1 diabetes mellitus (T1DM) and sought to develop a quantitative methylation-specific polymerase chain reaction (qMSP) assay for circulating beta cell DNA to monitor the loss of beta cells. Methylation-specific primers were designed to interrogate two or more CpG in the same assay. The cloned mouse *Ins2* gene was methylated *in vitro* and used for development of the qMSP assay. We found the qMSP method to be sensitive and specific to differentiate between insulin-producing cells and other tissues with a detection limit of 10 copies in the presence of non-specific genomic DNA background. We also compared different methods for data analysis and found that the Relative Expression Ratio method is the most robust method since it incorporates both a reference value to normalize day-to-day variability as well as PCR reaction efficiencies to normalize between the methylation-specific and bisulfite-specific components of the calculations. The assay was applied in the streptozotocin-treated diabetic mouse model and detected a significant increase in circulating beta cell DNA before the rise in blood glucose level. These results demonstrate that this qMSP assay can be used for monitoring circulating DNA from insulin-producing cells, which will provide the basis for development of assays to detect beta cell destruction in early T1DM.

## Introduction

Type 1 diabetes mellitus (T1DM) results from the autoimmune destruction [1], [2] of the insulin-producing beta cells by localized inflammation around the pancreatic islets involving cytotoxic T cells [3], [4] anti-islet antibodies [5], and antigen presenting cells [6]. The onset of metabolic dysregulation in T1DM occurs after the autoimmune destruction of the majority of beta cells, with loss estimates ranging between 60% and 80% of the total beta cell mass [7], [8]. Several studies have identified biomarkers for disease risk, such as anti-islet antibodies [9] and HLA genotyping [10], but a method of monitoring the underlying basis of the disease, namely the destruction of the beta cells, might allow early detection of the disease and also provide mechanistic assessment of new therapeutic interventions.

Lately, several clinical assays have been developed to monitor cell death *in vivo* based on the detection of nucleic acids that are released into the circulation by dying cells [11], [12]. These molecules can be detected by specific PCR-based assays [13], [14] thus providing a new approach for noninvasive assessment of the patient's status. In particular, DNA has a resident time in circulation from several weeks to more than a month, and many diagnostic tests have been developed based on circulating DNA molecules [15], [16], even some which differentiate DNA methylation patterns [17], [18]. The critical factor in these tests is the identification of a cell-specific nucleic acid biomarker for the disease.

Previous studies reported that detection of methylation of CpG islands showed a great promise as a biomarker for early detection of cancer [19], [20]. We have recently investigated the methylation patterns of both the mouse insulin (*Ins2*) and human insulin genes and found that there are three and nine CpG sites, respectively, in the promoters that are differentially methylated between insulin-producing cells and other adult tissues [21], and other portions of both these genes also exhibit tissue-specific methylation. We hypothesize that the beta cell-specific DNA methylation pattern might be detected in circulation of T1DM following the autoimmune destruction of beta cells and thus allow early detection of the onset and progression of the disease.

In this study we developed a sensitive, specific and quantitative methylation-specific PCR (qMSP) assay for circulating beta cell DNA and use it to monitor the development of diabetes in a mouse model of T1DM.

## Materials and Methods

### Ethics Statement

Animals used in this study received high quality animal care consistent with the Public Health Service Policy on Humane Care and Use of Laboratory Animals, the Guide for the Care and the Animal Welfare Act. The animal care facility at the City of Hope has been approved by the National Institutes of Health, registered with the U.S. Department of Agriculture, and fully accredited by the Association for Assessment and Accreditation of Laboratory Animal Care International (AAALAC). The study was approved by the City of Hope Institutional Animal Care and Use Committee (IUCAC# 07031). All surgery was performed under isoflurane anesthesia, and all efforts were made to minimize suffering. The experiments were designed to utilize the minimum number of animals required to obtain valid results.

### Animals

NOD/scid and BALB/c mice were obtained from The Jackson Laboratory and maintained under specific pathogen-free conditions. NOD/scid mice age 8 to 10 weeks old received intraperitoneal injections of 50 mg streptozotocin (STZ)/kg body weight on three consecutive days to induce diabetes. Blood glucose levels were measured pre- and post-STZ treatment at days 1, 2, 5, 6, 7, 14, and 35. At designated time points, about 200 µl blood was collected from each mouse for DNA purification and bisulfite conversion then used as a template for qMSP.

### Isolation of genomic DNA

Genomic DNA (gDNA) was obtained from NOD/scid mice (liver, pancreas, and blood), BALB/c mice (liver, spleen, kidney, brain, muscle, blood and beta cell fraction), and mouse NIT-1 insulinoma cells (ATCC, Manassas, VA) using the ZR genomic DNA kit (Zymo Research, Orange, CA) for cloning of the *Ins2* region and for studying tissue-specific methylation patterns of mouse *exon2*. Pancreatic beta cells were enriched to 60% of the total cell population as previously described [21]. Briefly, purified mouse islets were dispersed on-enzymatically and the resultant single cell suspension sorted by flow cytometry based on autofluorescence of the beta cells.

### PCR cloning of a fragment from mouse insulin gene

The 842 bp fragment of the mouse insulin gene (*Ins2*) from −480 to +362, containing nine CpG sites at positions −414, −182, and −171 in the promoter, +14 in exon 1, +121 in intron 1, and at +190, +310, +337, and +340 in exon 2, was PCR amplified from mouse gDNA using primers Ins2-pro-For and Ins2-pro-Rev (Table 1) and high-fidelity thermophilic DNA polymerase (Deep Vent DNA polymerase, NEB). The PCR product was cloned into pCR2.1-TOPO plasmid vector using the TOPO-TA cloning kit (Invitrogen). The cloned sequence was confirmed by DNA sequencing using M13F and M13R primers. The sequencing was performed by the DNA sequencing core facility in the Beckman Research Institute of City of Hope.

**Table 1:** Oligonucleotides used in this study.

	Designation	Sequence
**Primers for cloning the ** ***Ins2* fragment**		
1	Ins2-pro-For	5′-GAGCTCGGACCATTAAGTGCCTTGCTGCCT-3′
2	Ins2-pro-Rev	5′-CCATGGCTTGTGGGTCCTCCACTTCACG-3′
**Primers for methylation mapping of exon 2**		
1	Ins2-exon2-For	5′-TTTTTGTTATTTTTAATTTAGTTTATTTTTTAGGTTATTG-3′
2	Ins2-exon2-Rev	5′-ACAAAACTCACCTTATAAATCCTCCACTTCACA-3′
**Primers for qMSP and qBSP**		
P1	Promoter-bisulfite-For	5′-GTTTGGATTATTAAGTGTTTTGTTGTTTG-3′
P2	INS2-bisulfite-Rev1	5′-ACCCACTAAAAAAAATACCTTCCTACTTAC-3′
P3	INS2-bisulfite-Rev2	5′-AACTTATAAATCCTCCACTTCACAACA-3′
P4	1F Bisulf 5′	5′-TTTATTTTTGAGAGAGAGTTGGGGATTT-3′
P5	2F Bisulf 5′	5′-GATTTTAATTATTTTAGGATTAAGTAGAGGTGTTGAT-3′
P6	1R bisulf 5′ rev	5′-CTACCTAATAGTACAATACTAAATCTACAAAAAACA-3′
P12	BS-INS2 pro-For1	5′-GCAGGTTTTTATTTTTGAGAGAGAGTTGGGGATTT-3′
P13	BS-INS2 pro-Rev1	5′-CCTGCCAAACACTTCCCTAATACTAAATCTACAAAAAACA-3′
P16	BS-Pro-For	5′- CAGGTTTTTATTTTTGAGAGAGAGTTGGGGA-3′
P17	BS-Pro-Rev	5′- CCTGCCAAACACTTCCCTAATACTAAATCTACAAA-3′

Underline indicates extra nucleotides added at 5′-end of the primer.

### *In vitro* methylation of the *Ins2* fragment

The cloned *Ins2* fragment in pCR2.1-TOPO plasmid was methylated using M.*SssI* CpG methyltransferase (New England Biolabs, Ipswich, MA), which methylates all cytosine residues within the double-stranded dinucleotide recognition sequence CpG [21]. One microgram plasmid DNA was methylated using 10 U of M.*SssI* CpG methyltransferase, or in a parallel control reaction, mock-methylated in the absence of enzyme. Methylated and mock-methylated cloned plasmids were confirmed by *Hpa*II restriction enzyme digestion (New England Biolabs, Ipswich, MA). *Hpa*II is a methylation-sensitive restriction endonuclease that cleaves DNA at CCGG sequences when the internal cytosine residue is non-methylated on both strands.

### Bisulfite treatment

Methylated and unmethylated cloned plasmids or gDNA samples were treated with EZ DNA methylation-gold kit (Zymo Research, Orange, CA) according to the manufacturer's recommendation. The mouse *Ins2* gene was then amplified with pairs of specific primers (Table 1) by HotStarTaq polymerase (QIAGEN, Valencia, CA) according to the manufacturer's instructions.

### Quantitative methylation-specific PCR assay (qMSP)

Quantitative MSP was performed with a 7500 Real-Time PCR System (Applied Biosystems, Foster City, CA). Each reaction contained 15–25 ng of bisulfite-treated DNA as a template, 12.5 µl QuantiTect SYBR Green PCR (QIAGEN, Valencia, CA) and 500 nM each forward and reverse primers (Table 1) in a total volume of 25 µl. Thermal cycling was initiated with an enzyme activation step of 15 min at 95°C, followed by 40 cycles of 94°C for 15 s, 55°C for 30 s, 64°C for 30 s, 68°C for 30 s and, 72°C for 60 s. No-template controls were included in each run as negative controls to control for contamination during reactions. The quantification cycle (C_q_) [22] was determined for each reaction with methylation-specific primers (MSP) and bisulfite-specific primers (BSP) and the ratio of unmethylated to total amplifiable bisulfite-treated DNA was calculated by one of the following methods.

1. The ΔΔC_q_ method [23] is commonly used for gene expression analysis and shows the relative difference between the template of interest and a control template in the target sample compared with a reference sample. It was adapted to qMSP as follows using the cloned *Ins2* gene as the reference sample:
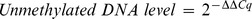


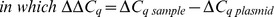


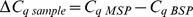


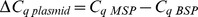
This equation assumes that the efficiency of the two reactions (MSP and BSP) is 100%. The C_q_ values were between 15 and 40.
2. The Relative Expression Ratio (RER) method was developed by Pfaffl [24] to account for differences in the efficiency of the PCR reactions. It was adapted to qMSP by using the cloned *Ins2* gene as the reference sample:
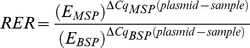
E_MSP_ is the efficiency of methylation-specific PCR, and E_BSP_ is the efficiency of bisulfite-specific PCR.
3. The Demethylation Index (DI) was developed by Akirav et al. [25] to determine the relative abundance of unmethylated beta cell DNA:
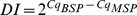

### Statistical analysis

Statistical significance between samples was tested with a two-tailed Student's *t*-test for unpaired values using GraphPad Prism 5 software. Statistical significance was stratified as a P-value of <0.05, <0.01, and <0.001. Statistical analysis of DNA methylation was done using QUMA (http://quma.cdb.riken.jp/) which performs a Fisher exact test on the methylation status of individual CpG sites. The statistical comparison of the Relative Expression Ratio and Demethylation Index was determined by Two-Way ANOVA.

## Results

### Beta cell-specific demethylation of exon 2 of the mouse *Ins2* gene

Our previous methylation mapping results of the mouse *Ins2* promoter revealed three CpG dinucleotide sites that are located at positions −414, −182, and −171 bp relative to the transcription start site (TSS) and these CpG sites have a specific methylation pattern in insulin-producing pancreatic beta cells and NIT-1 mouse insulinoma cells compared to other tissues [21].

**Figure 1:**
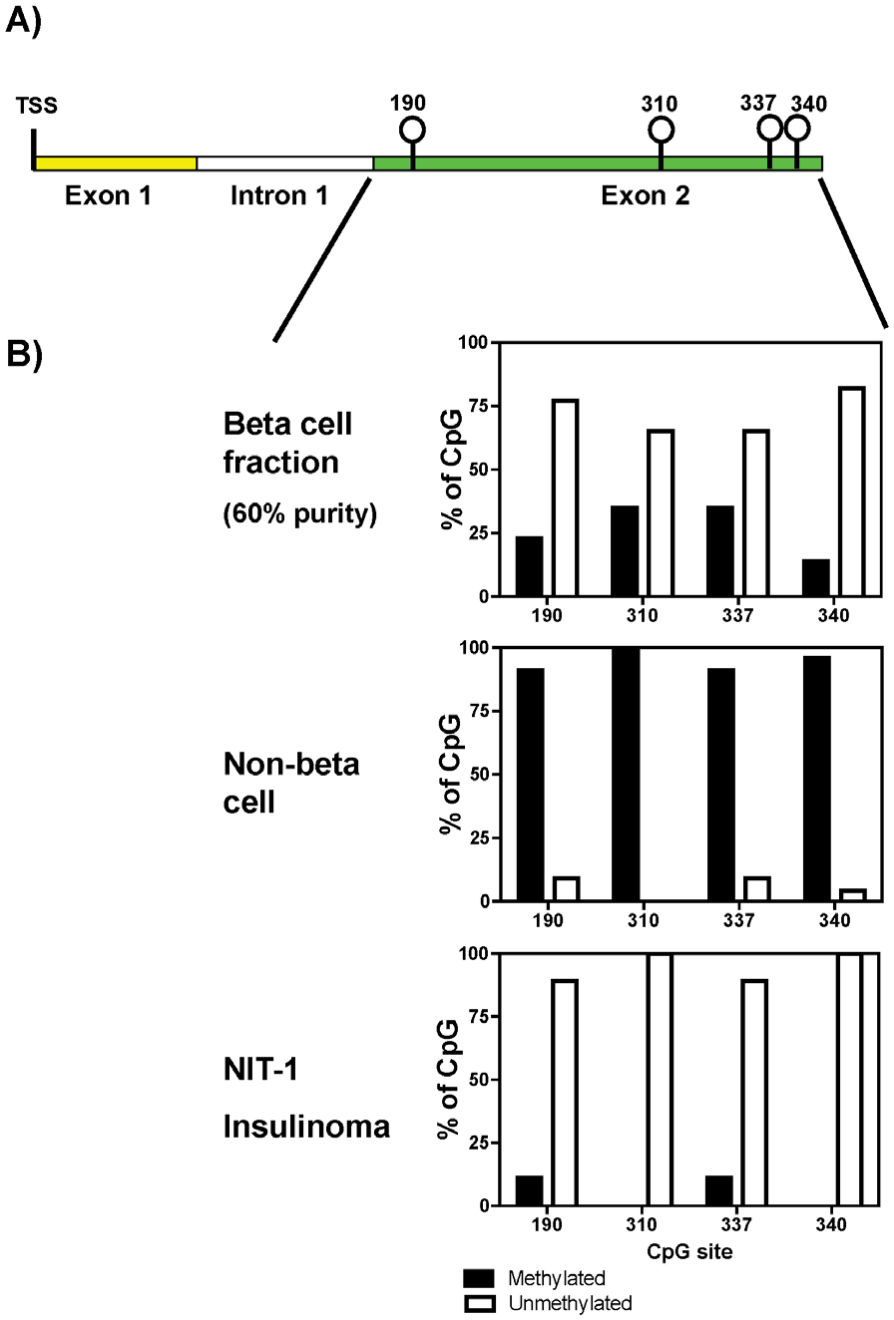
Beta cell-specific demethylation of exon 2 of the mouse *Ins2* gene. A) Schematic illustration of the mouse *Ins2* gene with exon 1 (yellow), intron 1 (white), and exon 2 (green) with the positions of the CpG sites indicated. B) Methylation pattern of mouse *Ins2* exon 2 showing the percentage of methylated (solid box) and unmethylated (empty box) CpG sites obtained from the 60% mouse beta cell fraction [21], NIT-1 mouse insulinoma cell line, and other mouse tissues (liver, spleen, kidney, brain, muscle, and blood). Each pattern results from 17, 9, and 23 clones of pancreatic beta cell fraction, NIT-1, and other tissues, respectively. TSS indicates the transcription start site.

To determine whether this tissue-specific methylation extended to other regions of the *Ins2* gene, gDNA samples from various mouse tissues were bisulfite-treated, amplified using primers specific for exon 2 (Ins2-exon2-For and Ins2-exon2-Rev; Table 1), and the methylation patterns determined by sequencing. Figure 1A shows the four CpG sites in the coding region of mouse exon 2 located at positions +190, +310, +337, and +340 bp relative to the TSS. The majority of the CpG sites in exon 2 were unmethylated in insulin-producing mouse beta cell-enriched fraction and insulinoma cells (Fig. 1B), but were predominantly methylated in the other tissues including liver, spleen, kidney, muscle, and blood. Furthermore, only in the beta cell fraction and insulinoma cells were there clones which were completely unmethylated. Altogether, there is a significant difference (p<0.0005) in the methylation frequency of each CpG site in exon 2 between beta cells and non-beta cells (Fig. 1B). Specifically, the CpG site at +190 was unmethylated in 13 of 17 clones (76%) from the enriched beta cell fraction versus 2 of 23 non-beta cells (9%), CpG at +310 was unmethylated in 11 of 17 beta (65%) versus 0 of 23 non-beta (0%), CpG at +337 was unmethylated in 11 of 17 beta (65%) versus 2 of 23 non-beta (9%), and CpG at +340 was unmethylated in 14 of 17 beta (82%) versus 1 of 23 non-beta (4%). These results demonstrate that exon 2 of the mouse *Ins2* gene exhibits a tissue-specific pattern of DNA methylation similar to what we previously reported for the mouse *Ins2* promoter.

### Rationale for selection of the primers that differentiate between methylated and unmethylated CpG

**Figure 2:**
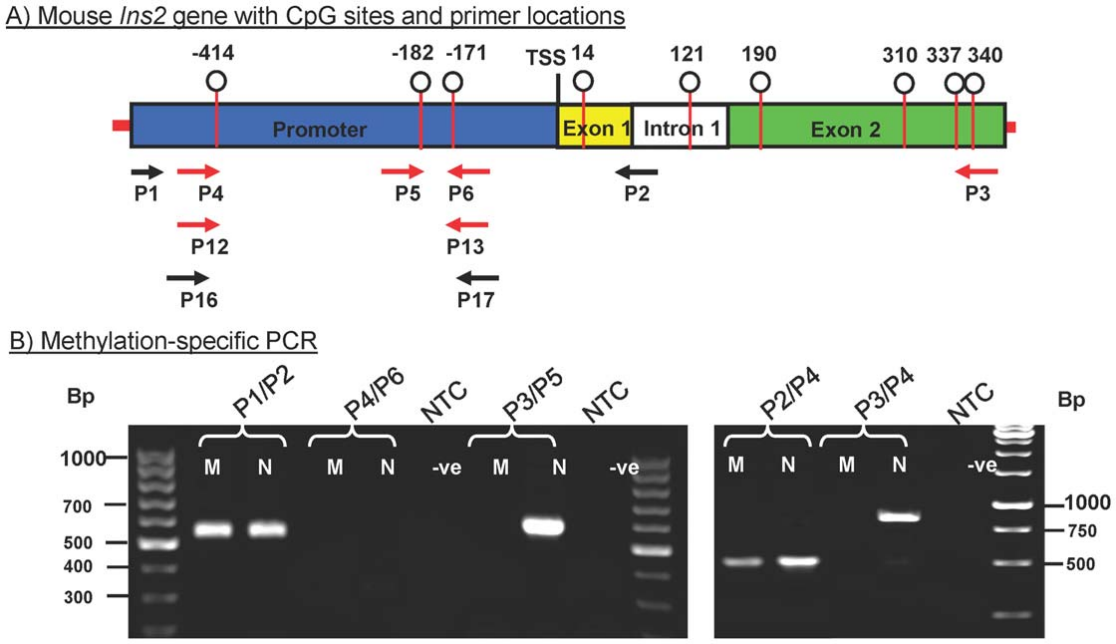
Rationale for selection of the primers that differentiate between methylated and unmethylated CpG. A) Schematic illustration of the mouse *Ins2* gene with promoter region (blue), exon 1 (yellow), intron 1 (white), and exon 2 (green) showing the positions of CpG sites and the primers used in this study. Black arrows represent the bisulfite-specific primers (BSP) that amplify both methylated and unmethylated DNA. Red arrows represent methylation-specific primers (MSP) that amplify unmethylated but not methylated DNA. B) Gel electrophoresis (3% agarose) of PCR products amplified by reactions using different primer sets and the cloned *Ins2* gene as template. The clone was methylated (M) or sham methylated (N) and bisulfite-treated prior to use in the reactions. NTC means non-template control. TSS indicates the transcription starting site.

To develop a method to discriminate between these methylation differences, methylation-specific primer pairs were designed for qMSP to have similar melting temperatures and of about 30 bases in length, and each primer was matched at the 3′-terminus with the bisulfite-treated unmethylated CpG site sequence. As shown in Figure 2A, there are 3 CpG sites in the promoter region and 4 in the exon 2 region and primers were designed to match the unmethylated sequences at positions +337 (P3), −414 (P4), −182 (P5), and −171(P6) (Table 1). Primer sets P4/P6, P3/P5 and P3/P4 are methylation-specific primer pairs that are able to distinguish the methylation status of specific CpG sites (MSP). They amplify fragments of 304 bp, 580 bp and 804 bp, respectively, from unmethylated but not methylated DNA (Fig. 2B). Additionally, a set of bisulfite-specific primers (P1/P2) was similarly designed to amplify all bisulfite-treated DNA regardless of methylation status (BSP), and therefore amplifies a 548 bp fragment from both methylated and unmethylated DNA (Fig. 2B). Combining the bisulfite-specific primer P2 with the methylation-specific primer P4 amplifies a fragment of 505 bp from both the methylated and unmethylated DNA (Fig. 2B) with only moderate methylation specificity. This is because the bisulfite-specific primer 2 is complementary to both the methylated and unmethylated template while primer 4 is a methylation-specific primer that only matches the unmethylated template.

**Figure 3:**
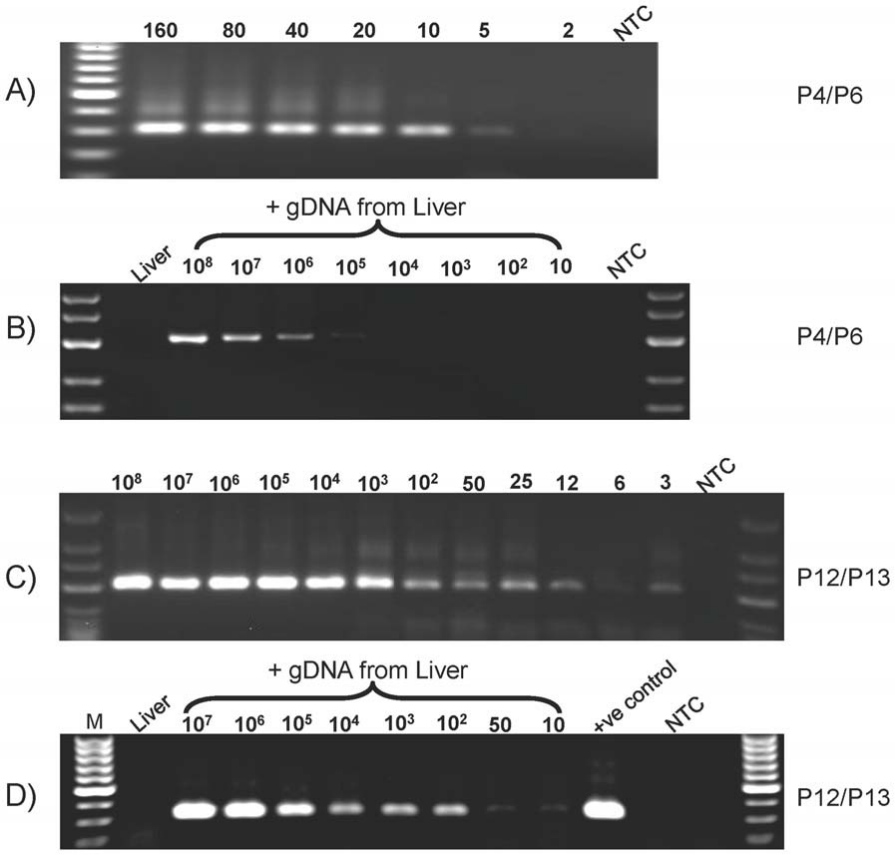
Effect of non-specific gDNA on the specificity and sensitivity of MSP. The unmethylated *Ins2* gene plasmid was diluted in the presence or absence of 500 ng non-specific gDNA, bisulfite-treated, and used as template for PCR. A) 160, 80, 40, 20, 10, 5, and 2 copies of plasmid without non-specific DNA analyzed by qMSP using primer set P4/P6. B) Serial dilutions ranged from 10^8^ to 10 copies of plasmid in the presence of non-specific gDNA analyzed by qMSP using primer set P4/P6. C) Serial dilution from 10^8^ to 3 copies of plasmid in the absence of non-specific gDNA analyzed by qMSP using primer set P12/P13. D) Range of serial dilutions from 10^7^ to 10 copies plasmid in the presence of non-specific gDNA analyzed by qMSP using primer set P12/P13. Mouse liver gDNA was used as non-specific DNA and NTC is the non-template control.

To analyze the specificity of these primer sets, the mouse *Ins2* gene was cloned, methylated or sham methylated *in vitro*, bisulfite-treated, and used as templates for PCR. Methylation mimics the state of the *Ins2* gene in non-beta cells, and the sham methylated template is like the *Ins2* gene in beta cells. The ability of the primer sets to distinguish methylated versus unmethylated templates was determined by PCR using increasing concentrations of the bisulfite-treated methylated or unmethylated plasmids. The primer set P4/P6 (Fig. 3A) detected as low as 2 copies of unmethylated plasmid, but the lower limit for methylated plasmid was one million copies, which calculates to an analytical specificity (i.e. detection of the appropriate target sequence rather than nonspecific targets) of >10^5^ [22], indicating that this primer set exhibited good sensitivity and specificity.

### Effect of genomic DNA background on the specificity and sensitivity of MSP

To evaluate the ability of the primer sets to detect unmethylated beta cell DNA in the presence of a large amount of non-beta cell gDNA such as would be found in blood samples, the methylation-specific primers (P4/P6) were used for amplification using serially diluted unmethylated DNA template in the absence (Fig. 3A) and presence (Fig. 3B) of a background of 500 ng of nonspecific gDNA from mouse liver added prior to bisulfite treatment. Comparing Figures 3A and 3B, the primer set (P4/P6) regularly detected as little as 2 copies of unmethylated template in the absence of non-specific DNA, but only 10^5^ copies of template (equivalent to 5×10^4^ beta cells) in the presence of non-specific DNA. Similarly, all of the primer pairs depicted in Figure 2B displayed a dramatic reduction in the sensitivity and selectivity with the addition of non-specific gDNA.

Based on these results, it appeared necessary to redesign the primers to provide better specificity and sensitivity. However, a major problem that arises in development of MSP assays is that the bisulfite treatment decreases the GC content and the complexity of the target sequence, which greatly restricts the design of the primers, which also must align their 3′ end with the CpG site being interrogated. In the case of the unmethylated insulin gene these limitations are further exacerbated by the requirement of a 3′ thymidine residue to match the bisulfite-treated unmethylated cytosine present in the beta cell-specific sequence, which further degrades the specificity. To overcome these problems and improve matching of primer GC content and annealing temperatures, the complementary regions of the primers were increased and short GC-rich sequences were added to the 5′ ends of primers P4 and P6 (Table 1, Fig. 2A), resulting in primers P12 and P13, respectively, which are specific for CpG sites at −414 and −171 (MSP). As before, the specificity and sensitivity of the redesigned primers were evaluated using serially diluted unmethylated DNA template in the absence (Fig. 3C) and presence (Fig. 3D) of non-specific gDNA. As shown in Figure 3C, this primer set exhibits dose-dependent amplification of a 316 bp fragment from 10^8^ to as little as 3 copies of unmethylated template alone (equivalent to 1.5 beta cells). Furthermore, as shown in Figure 3D, the redesigned primer set is largely unaffected by the presence of the non-specific DNA and is able to detect 10 copies of unmethylated template (equivalent to 5 beta cells), but does not produce a detectable signal from the non-specific mouse liver gDNA control (Fig. 3B and 3D “Liver”; equivalent to ≈10^5^ non-beta cells).

### Analytical performance of the quantitative methylation-specific assay (qMSP)

**Figure 4:**
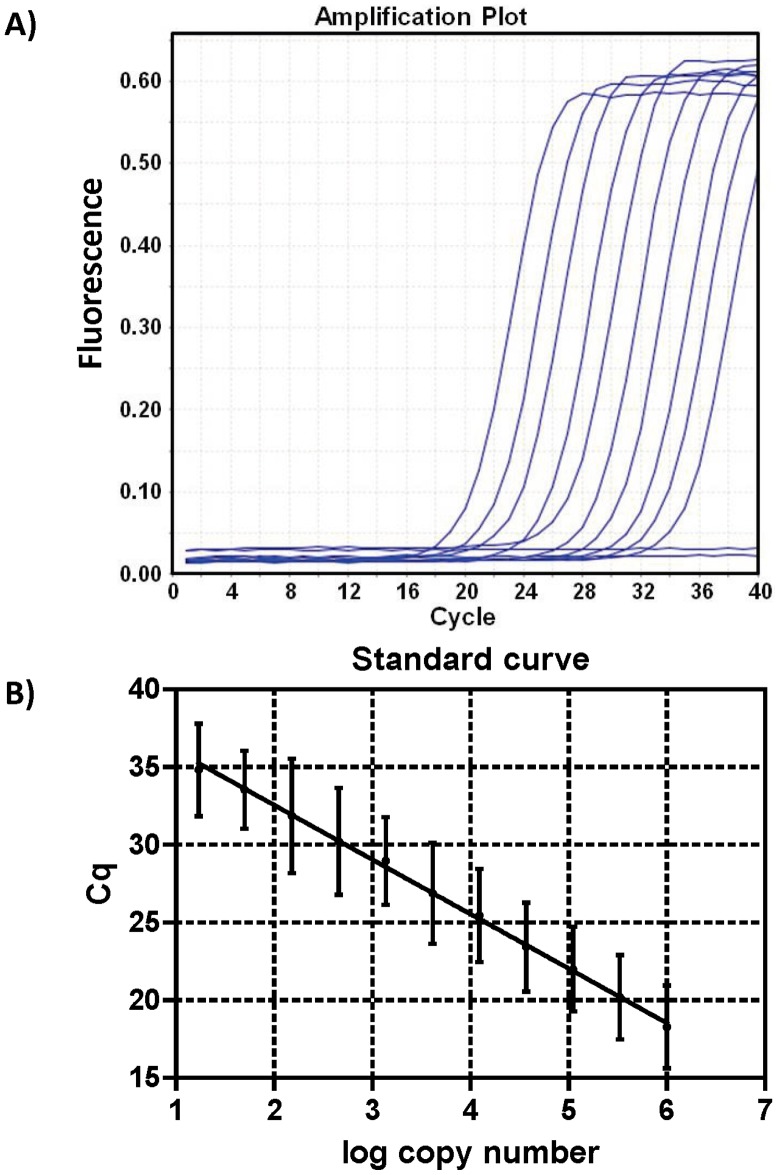
Analytical performance of qMSP with SYBR Green and methylation-specific primers. A) Amplification plot of a three-fold serial dilution of unmethylated plasmid showing the fluorescence versus cycle using P12 and P13 as methylation-specific primers. B) Standard curve plot showing C_q_ versus log copy number of three-fold serially diluted bisulfite-treated unmethylated plasmid and the data shown are the average of 12 repeats with standard deviation (SD).

For application to mouse blood samples, the P12/P13 primer set was used to develop a SYBR Green-based qMSP assay. Three fold serial dilutions of unmethylated plasmid (10^6^ copies to 17 copies) were used to prepare a standard curve (Fig. 4). Standard curve analysis revealed that the qMSP assay was linear over more than a 10^5^-fold range of template concentrations. Variation across the curve ranges from 8.5% to 14.6% with an average 11.4%±1.9 (Fig. 4B and Table 2). Table 3 shows that the standard curve parameters were highly reproducible (efficiency = 93.34%±8.9 SD, slope = −3.51±0.23 SD, R^2^ = 0.97±0.04 SD; n = 12 experiments).

**Table 2:** Statistical variation of qMSP standard curve.

Log Copy Number	Average C_q_ *	±SD	%CV
**1.2**	34.82	2.98	8.5
**1.7**	33.54	2.52	7.5
**2.2**	31.87	3.69	11.6
**2.7**	30.22	3.44	11.4
**3.1**	28.97	2.82	9.7
**3.6**	26.89	3.24	12.1
**4.1**	25.44	3.00	11.8
**4.6**	23.42	2.87	12.2
**5.0**	22.00	2.71	12.3
**5.5**	20.20	2.71	13.4
**6.0**	18.28	2.67	14.6

* n = 12; SD = standard deviation, %CV = percent coefficient of variation [(SD/Cq average) ×100].

**Table 3:** The amplification efficiency of qMSP and qBSP standard curves.

PCR type (Primer set)	Efficiency % ±SD	Slope ±SD	R^2^ ±SD
**MSP (P12/P13)**	93.34±8.92	−3.51±0.23	0.97±0.04
**BSP (P16/P17)**	91.47±5.62	−3.55±0.15	0.99±0.01

MSP = methylation-specific PCR, BSP = bisulfite-specific PCR, Slope = slope of the standard curve, R^2^ = the square of the correlation coefficient of the standard curve.

To normalize the results obtained with blood samples, a quantitative bisulfite-specific PCR assay (qBSP) was developed in parallel with the qMSP assay to quantify the total level of amplifiable bisulfite-treated gDNA in each sample. Primers P16 and P17 (Table 1, Fig. 2A) were designed to overlap the template sequences for the P12/P13 primer set but are not dependent on the methylation status of the CpG sites (BSP). The qBSP standard curve consisting of three-fold serial dilutions of methylated plasmid (10^6^ copies to 17 copies) exhibits linearity (efficiency = 91.47%±5.6 SD, slope = −3.55±0.15 SD, R2 =  0.99±0.01 SD; n = 4 experiments) over the entire dose range (Table 3).

### Detection of circulating beta cell-specific DNA by qMSP in mouse blood

**Figure 5:**
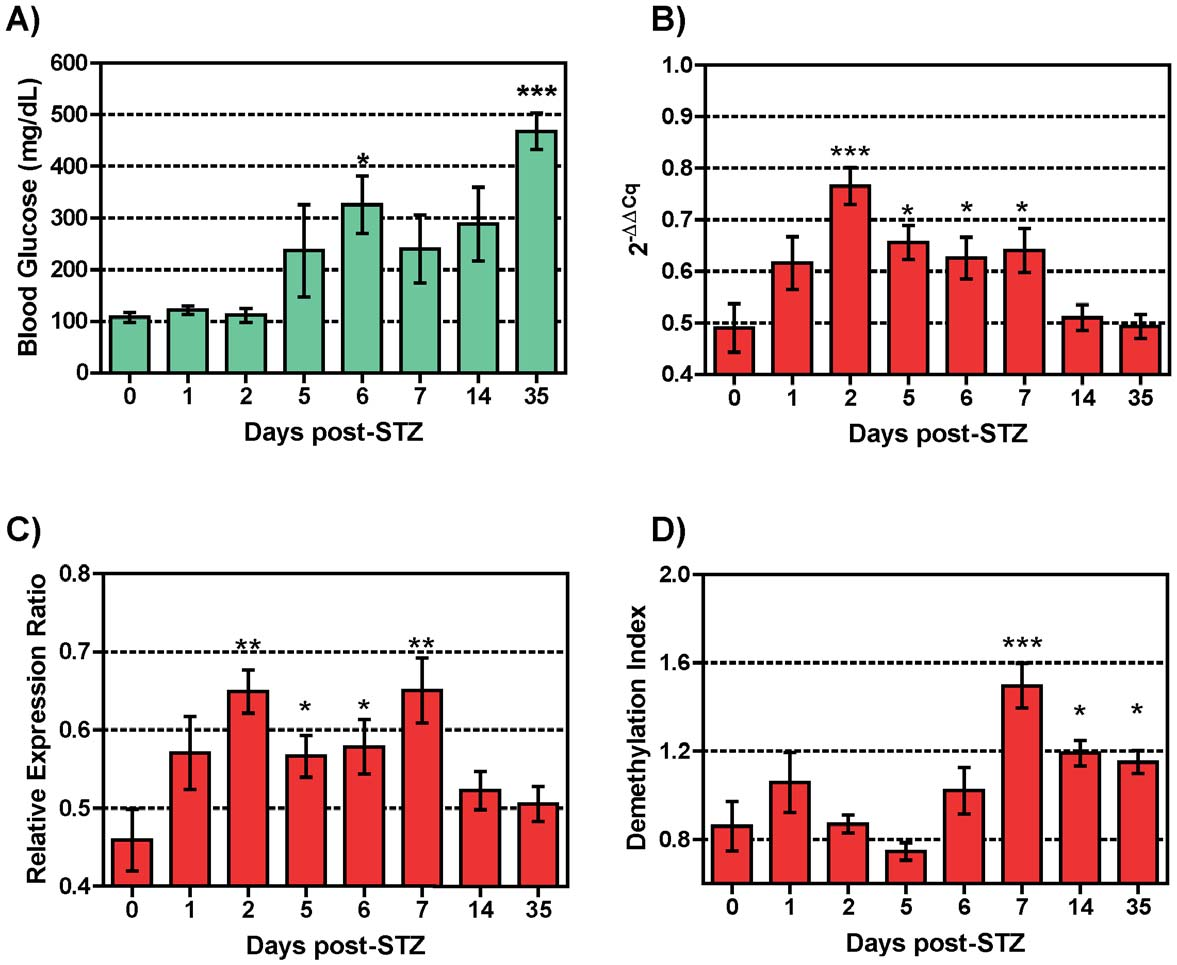
Quantification of circulating beta cell DNA in STZ-treated diabetic mice. NOD/scid mice were injected with STZ at days 0, 1, and 2, and blood was collected pre-treatment and post-treatment days 1, 2, 3, 5, 6, 7, 14, and 35. A) Blood glucose levels for untreated (n = 3) and STZ-injected (n = 34) NOD/scid mice were measured at days 1 (n = 6), 2 (n = 4), 5 (n = 4), 6 (n = 8), 7 (n = 4), 14 (n = 4) and 35 (n = 4) after injection. In parallel, qMSP was done using bisulfite converted gDNA obtained from the blood of untreated (n = 3) and STZ-treated mice at designated time points. Fold changes in demethylation are measured by calculation of ΔΔC_q_ (B), Relative Expression Ration (C) or Demethylation Index (D) for each sample using methylation-specific primers P12/P13 and bisulfite-specific primers P16/P17. The cloned *Ins2* gene was used for normalization and standardization of the results as described under Material and Methods. The data display the mean ± standard error (SEM) of three independent measurements. The statistical significance was calculated with the Student *t* test for unpaired values and significance level indicated by asterisks (*, P<0.05; **, P<0.01: *** P<0.001).

To evaluate the ability of the assay to detect beta cell destruction *in vivo*, NOD/scid mice were treated with the beta cell-specific toxin streptozotocin (STZ) for three consecutive days starting at day 0 and blood samples were collected at days 1, 2, 5, 6, 7, 14, and 35 post-treatment. STZ-treatment resulted in a significant decrease in islet area (Fig. S3) and a notable increase in blood glucose levels after 5 days post-treatment which reached significance at day 6 (Fig. 5A).

Genomic DNA was also isolated from the collected blood and then bisulfite-treated and used as a template for qMSP. After amplification, the qMSP and qBSP reactions were electrophoresed on a 3% agarose gel as shown in Figure S1, to confirm the correct size of the PCR products. In addition, sequence analysis confirmed the identity of the PCR products (Fig. S2).

Using the standard ΔΔC_q_ method of analysis, the methylation-specific primers detect an increase in the level of circulating unmethylated DNA resulting from islet destruction one day post-treatment, reaching significance on day 2 (Fig. 5B), well before the rise in blood glucose level (Fig. 5A). The level of circulating beta cell DNA remains significantly higher than control NOD/scid mice on days 2, 5, 6 and 7 (p = 0.0004, p = 0.0126, p = 0.0449, and p = 0.0331, respectively) after STZ treatment, only returning to pre-treatment levels by day 14. In summary, these results demonstrate the ability of qMSP to detect unmethylated DNA resulting from beta cell death in the blood of the STZ-treated mouse model.

### Comparison of methods of analysis of qMSP data

In the report by Akirav et al. [25] the results of the qMSP assay were analyzed using the “Demethylation Index” (DI), which is based on the difference between the level of the bisulfite-specific PCR and the methylation-specific PCR, and assumes an equal efficiency of both reactions of 100%. We initially investigated this method for analysis of our data but found high day-to-day variability. Therefore, two other methods of analysis were evaluated: the ΔΔC_q_ method, which is commonly used for analysis of relative gene expression, and the Relative Expression Ratio (RER) [24], which is similar to ΔΔC_q_ but also takes into account the differences in the efficiency of the PCR reactions (see Materials and Methods for details). In each of the three methods, the total amplifiable template as represented by the qBSP result (reference value) was used to normalize the qMSP data for each sample. For ΔΔC_q_ and RER, the result using the unmethylated *Ins2* plasmid template was included as the control sample in the calculations for each sample set. Analysis of the diabetic mouse data shows that the ΔΔC_q_ and RER calculation methods (Fig. 5C and Table 4) give similar results, with a rise in circulating beta cell DNA relative to total amplifiable DNA by day 1 and increasing to a significant level by day 2. In both these data sets the beta cell DNA levels remain significantly elevated until day 7 and then decline to basal levels, though the animals remain hyperglycemic. By contrast, the DI calculations (Fig. 5D and Table 4) do not exhibit a clear pattern, but rise to significance on day 7 and remain elevated.

**Table 4:** Comparison of the average and standard deviation of three methods of quantification of unmethylated DNA levels using qMSP.

		Relative Expression Ratio		2^−ΔΔ Cq^		Demethylation Index	
Days post-STZ	n	Average (± SD)	P value*	Average (± SD)	P value*	Average (± SD)	P value*
0	9	0.459 (0.118)	NA	0.490 (0.141)	NA	0.860 (0.336)	NA
1	12	0.571 (0.162)	0.098	0.616 (0.176)	0.095	1.058 (0.468)	0.296
2	8	0.649 (0.079)	0.002	0.766 (0.101)	0.0004	0.870 (0.114)	0.963
5	8	0.566 (0.075)	0.044	0.656 (0.092)	0.013	0.747 (0.112)	0.378
6	16	0.579 (0.135)	0.039	0.626 (0.157)	0.045	1.021 (0.404)	0.327
7	8	0.650 (0.118)	0.005	0.640 (0.120)	0.033	1.496 (0.287)	0.001
14	8	0.523 (0.069)	0.204	0.510 (0.070)	0.722	1.191 (0.163)	0.023
35	8	0.506 (0.064)	0.340	0.493 (0.064)	0.962	1.150 (0.149)	0.040

n is the number of replicates at each time point and represents data from at least 4 mice.

* P-value calculated by Student's *t*-test for the difference between each time point and pre-treatment value. SD is the standard deviation. N/A = not applicable.

**Figure 6:**
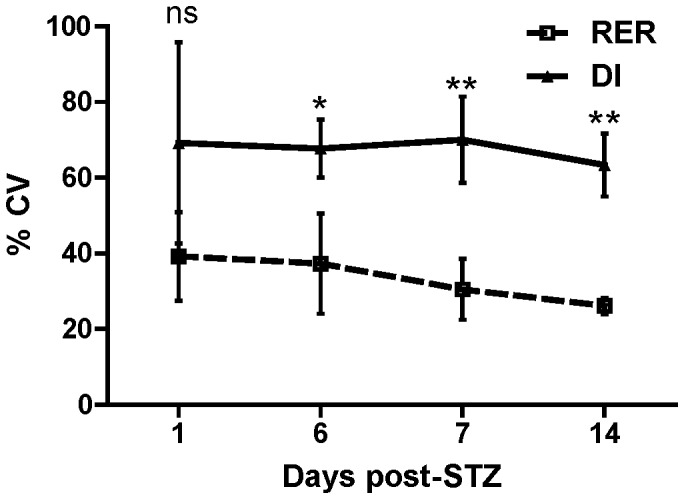
Reproducibility of the qMSP assay. The variability of the qMSP results at different time points as analyzed using Relative Expression Ratio (RER) compared with Demethylation Index (DI) at days 1, 6, 7, and 14 post-STZ treated mice. The data display the percent coefficient of variation (%CV) with standard deviation (SD) of the variation at each point representing data from at least 4 mice. The statistical significance at each time point was calculated by Two-Way ANOVA to compare RER and DI and the significance level indicated by asterisks (*, P<0.05; **, P<0.01). ns =  non significant.

To evaluate the merits of these methods, the reproducibility of the DI method was compared with RER at days 1, 6, 7, and 14 post-STZ, and the variability of the assay as expressed as the coefficient of variation was determined at different time points. The ΔΔC_q_ method was not included in the analysis since the data was very similar to RER, and RER additionally includes PCR efficiency in the calculation. As shown in Figure 6, the variability of the DI method is significantly higher than RER on days 6, 7, and 14, and the mean is higher on day 1 but does not reach the level of significance. Moreover, Table 5 shows the inter-assay reproducibility (day-to-day reproducibility) [26] is better with RER compared with DI. Specifically, in case of RER the coefficient of variation ranged from 21.64% to 38.72 whereas for DI it ranged from 60.17% to 74.85%. These results indicate that RER provides a more reproducible method for analysis of the qMSP results than DI, a conclusion which is expected since RER incorporates both a reference value and PCR reaction efficiencies to normalize day-to-day variability and to normalize between the qMSP and qBSP components.

**Table 5:** Inter-assay reproducibility of the qMSP assay.

	Replicate assay				
	Assay 1	Assay 2	Assay 3	Average (± SD)	% CV
**Relative Expression Ratio**					
Day 1	0.610	0.541	1.068	0.739 (0.286)	38.72
Day 6	0.568	0.568	1.033	0.723 (0.269)	37.17
Day 7	0.650	0.654	0.984	0.763 (0.192)	25.13
Day 14	0.523	0.647	0.807	0.659 (0.143)	21.64
**Demethylation Index**					
Day 1	1.398	0.408	0.491	0.766 (0.549)	71.67
Day 6	1.138	0.423	0.454	0.672 (0.404)	60.17
Day 7	1.496	0.504	0.410	0.803 (0.601)	74.85
Day 14	1.191	0.477	0.334	0.667 (0.459)	68.83

Replicate assays were done on three separate days for each time point, and each assay represents the mean data for at least 3 mice. SD is the standard deviation, and % CV is the percent coefficient of variation [(SD/average) ×100].

## Discussion

Type 1 diabetes mellitus (T1DM) is an autoimmune disorder in which cytotoxic T lymphocytes identify and destroy insulin producing beta cells in the pancreas. This autoimmune destruction is silent and more often than not diagnosed after the presentation of devastating clinical symptoms [27]. In this study, we demonstrate a highly sensitive and quantitative MSP assay for the detection of circulating beta cell DNA in peripheral blood from STZ induced diabetic mice and use it to monitor the onset of diabetes.

Quantitative MSP is sensitive and specific for detection of rare DNA [28], and is able to differentiate between methylated and unmethylated DNA by using oligonucleotides whose 3′-ends match the methylation status of specific CpG sites in a bisulfite-treated template. Using an *in vitro* model based on unmethylated plasmid, we detected the equivalent of 5 beta cells in the presence of 10^5^ other mouse cells. Mouse blood contains ≈10^7^ nucleated cells/ml [29], and a 25 gm mouse has about 2 ml of total blood volume (80 ml/kg) [30] and so the total number of circulating nucleated cells for each mouse ≈2×10^7^. From this calculation, we can determine that the assay can detect DNA obtained from 1000 beta cells (equivalent to one islet) distributed in the entire blood volume of the mouse [31], [32]. It has been reported that changes in blood glucose result after the loss of more than 65% of the islets [7], so we believe that this assay will be more than sensitive enough to monitor the onset of beta cell destruction prior to the metabolic manifestation.

An important aspect of this assay is the tissue-specific patterning of DNA methylation. This provides accurate identification of the cell source of the circulating DNA, and therefore the underlying pathology associated with changes in the signal. We previously reported that the mouse *Ins2* gene promoter displays a tissue-specific methylation pattern unique to beta cells, and in this study we extended this to *Ins2* exon 2 and found that it also exhibits a tissue-specific methylation pattern, and therefore would likely be another good region for targeting the assay. We believe that careful mapping of the pattern of the target gene in several tissues is critical to reliable application of this methodology to clinical diagnostics.

An interesting observation we made while developing these assays was that the specificity and sensitivity was greatly affected by the presence of non-specific DNA. Primer pairs that exhibited excellent specificity to the template alone showed no specificity in the presence of excess non-specific DNA. However, the positioning and design of the primers is limited to the region adjacent to each particular CpG site. To overcome this problem we redesigned the primers to include an untemplated “clamp” on the 5′-ends to better match primer melting temperatures and increase the percentage of GC content. The presence of this clamp substantially improved the specificity and sensitivity of the assay. We have yet to test this approach extensively, but feel that it may be a useful in the development of other diagnostic MSP assays.

Current methods used for diagnosis of T1DM measure metabolic dysfunction, such as blood glucose or HbA1c, or measure the immune response, such as autoantibodies [33] or immune cell populations [34], [35], both of which indicate beta cell death indirectly but do not directly measure the beta cell destruction. By monitoring the appearance of beta cell DNA in blood, we are measuring the extent of beta cell loss leading to diabetes. We were able to detect acute beta cell destruction in STZ induced diabetic mouse model by the emergence of unmethylated DNA one day after STZ treatment, while hyperglycemia was not observed until four days later, demonstrating our method's ability to detect beta cell death before a detectable increase in blood glucose level. This early detection method could provide a window of opportunity for effective intervention to slow or prevent the progression of the disease.

Akirav et al [25] have reported a method for monitoring beta cell destruction *in vivo* by using primers that are specific for DNA methylation patterns in beta cells, and have detected circulating unmethylated DNA from beta cell death in serum of mice by quantitative nested PCR. Actually this method is to some extent similar to our method, but there are some distinct differences that we believe will affect translation to a clinical setting. First, the authors included a gel purification step for the first PCR product to be used as a template for second reaction of nested PCR. Recovery of product from gel purification can be quite variable and may also introduce PCR inhibitors which will affect the downstream reactions. It will also be difficult to implement in clinical application. Secondly, the second PCR reaction uses one methylation-specific primer with one bisulfite-specific primer, meaning the assay only probes one CpG site and therefore is not fully methylation-specific PCR. We demonstrate the limitation of this approach in Figure 2B in which the methylation-specific primer P4 was paired with the bisulfite-specific primer P2. The reaction exhibits only moderate methylation-specificity compared with primer sets which are specific for two methylation sites. If a nested PCR procedure is implemented, it would probably be better to utilize methylation-specific primers in both reactions to increase the specificity of the assay.

Akirav et al. [25] also introduced a new calculation method called the “Demethylation Index” (DI) to quantify beta cell DNA in blood samples. While they reported that this method was effective for detecting signals in both mouse and human blood samples, we noted that it did not provide the means for sample normalization across patients and across different laboratories. We initially used DI for analysis of our results but found that in our hands there was high variability in the data. Therefore we investigated the use of two calculation strategies commonly used for comparative gene expression analysis, namely ΔΔC_q_ [23] and a similar method called the Relative Expression Ratio method [24] which takes into account the efficiency of both the reference and target PCR reactions, which in the case of gene expression makes the results more comparable between different experiments and laboratories [22]. We adapted these methods to qMSP by including a plasmid control in each assay to normalize for day-to-day differences in PCR efficiency. In our hands, this provided better standardization of the signals and was critical to interpretation of the results by improving day-to-day reproducibility (Table 5). By contrast, the DI results did not seem to correlate with onset of diabetes in these animals. We were surprised by clear differences in the results since all three data sets utilized the same MSP and BSP data, but the first two included the plasmid data as a reference control for individual PCR assays done on different animals at different times, whereas the latter only included the internal qBSP control. We would also point out that we measured the efficiency of both the qMSP and the qBSP reactions (Table 3) and they are less than 100% and substantially different from each other, so it is important to take this information into account in the data analysis. From these results we believe that this will be an important consideration as we move the human assay into the clinical studies and compare data from different patients over time. It is our opinion from this analysis that RER may be the best of these methods since it incorporates both a reference value to normalize day-to-day variability as well as PCR reaction efficiencies to normalize between the MSP and BSP components of the calculations.

In conclusion, this study demonstrates a qMSP assay with significant specificity and selectivity for the detection of circulating beta cell DNA. The demonstrated sensitivity and specificity of this assay will allow it to be used for a more accurate and early detection of beta cell death and early onset of diabetes.

## Supporting Information

Figure S1 **PCR products of qMSP and qBSP amplified from mouse blood.** Gel electrophoresis (3% agarose) showing products from PCR using methylation-specific primers (MSP, A) and bisulfite-specific primers (BSP, B) and circulating DNA from non-treated NOD/scid mice (blood control) and NOD/scid mice at days 1, 2, 5 and 6 after injection with STZ as detailed in Material and Methods. DNA isolated from mouse pancreas and from NIT-1 insulinoma was used as positive controls. NTC means non-template control.
(TIFF)

Figure S2 **Alignment of the sequences of the MSP and BSP products.** DNA from STZ-treated mice and from unmethylated cloned *Ins2* fragment were bisulfite-treated and PCR amplified as detailed in Materials and Methods using, A) MSP primer set P12/P13, and B) BSP primer set P16/P17 and the products were sequenced. PCR sequences were aligned with the expected sequence using MultAlin (http://multalin.toulouse.inra.fr/multalin/).
(TIFF)

Figure S3 **Significant reduction of islet area in STZ-treated diabetic mouse model.** Pancreata removed from untreated control mice and STZ-treated mice that developed diabetes (n = 5 each) were fixed in 10% buffered formalin solution, embedded in paraffin and sectioned. The tissue sections were stained with hematoxylin and eosin (HE) using Tech-mate 1000 autostainer (Ventana, Tucson, AZ) by the City of Hope Anatomical Pathology Core. Stained sections were covered with Vectashield (Vector Laboratories, Burlingame, CA) and visualized using an Olympus IX51 fluorescent microscope equipped with an infinity 2 camera (Olympus America, Melville, NY). Pictures were captured using Infinity Analyze acquisition 5.0 software (Lumenera Corporation, Ottawa, Canada. A) Hematoxylin and eosin staining of pancreatic section of untreated control (left) and STZ-treated diabetic (right). Islets are indicated with arrows. B) The percent pancreatic islet area was quantified from histological sections of untreated control mice and STZ-treated mice using Fiji software (http://fiji.sc/wiki/index.php/Fiji). The data display the mean ± standard deviation (SD). The statistical significance was calculated with the Student *t* test for unpaired values and significance level indicated by asterisks (*** P<0.001).
(TIFF)
