# Supplementary figures and images for: Development of a Quantitative Methylation-Specific Polymerase Chain Reaction Method for Monitoring Beta Cell Death in Type 1 Diabetes
Source: PLoS One. 2012 Oct 29;7(10):e47942. doi: 10.1371/journal.pone.0047942 (PMC3483298; doi:10.1371/journal.pone.0047942)

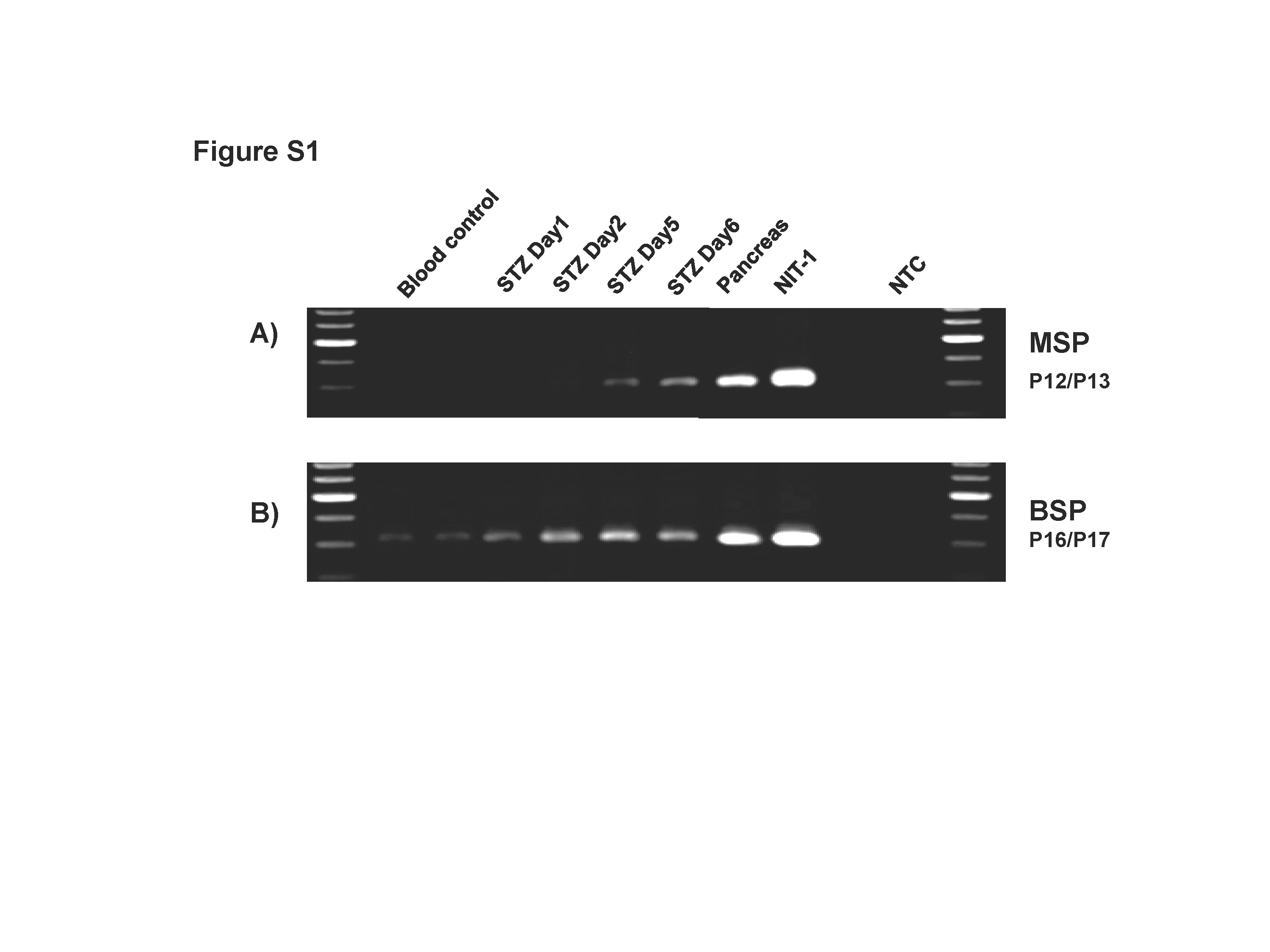

Supplement: Figure S1 — PCR products of qMSP and qBSP amplified from mouse blood. Gel electrophoresis (3% agarose) showing products from PCR using methylation-specific primers (MSP, A) and bisulfite-specific primers (BSP, B) and circulating DNA from non-treated NOD/scid mice (blood control) and NOD/scid mice at days 1, 2, 5 and 6 after injection with STZ as detailed in Material and Methods. DNA isolated from mouse pancreas and from NIT-1 insulinoma was used as positive controls. NTC means non-template control. (TIFF) [file pone.0047942.s001.tiff]

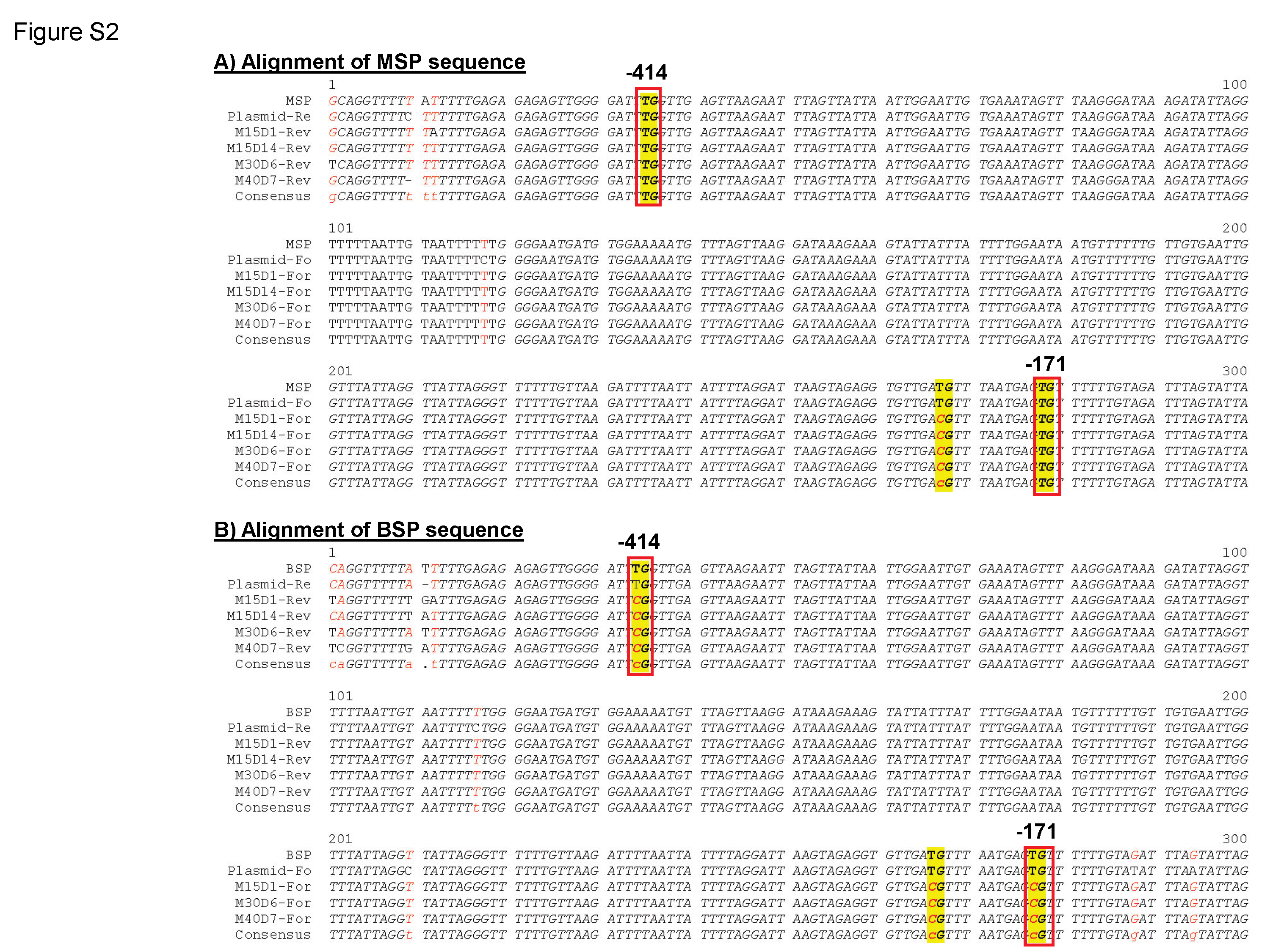

Supplement: Figure S2 — Alignment of the sequences of the MSP and BSP products. DNA from STZ-treated mice and from unmethylated cloned Ins2 fragment were bisulfite-treated and PCR amplified as detailed in Materials and Methods using, A) MSP primer set P12/P13, and B) BSP primer set P16/P17 and the products were sequenced. PCR sequences were aligned with the expected sequence using MultAlin (http://multalin.toulouse.inra.fr/multalin/). (TIFF) [file pone.0047942.s002.tiff]

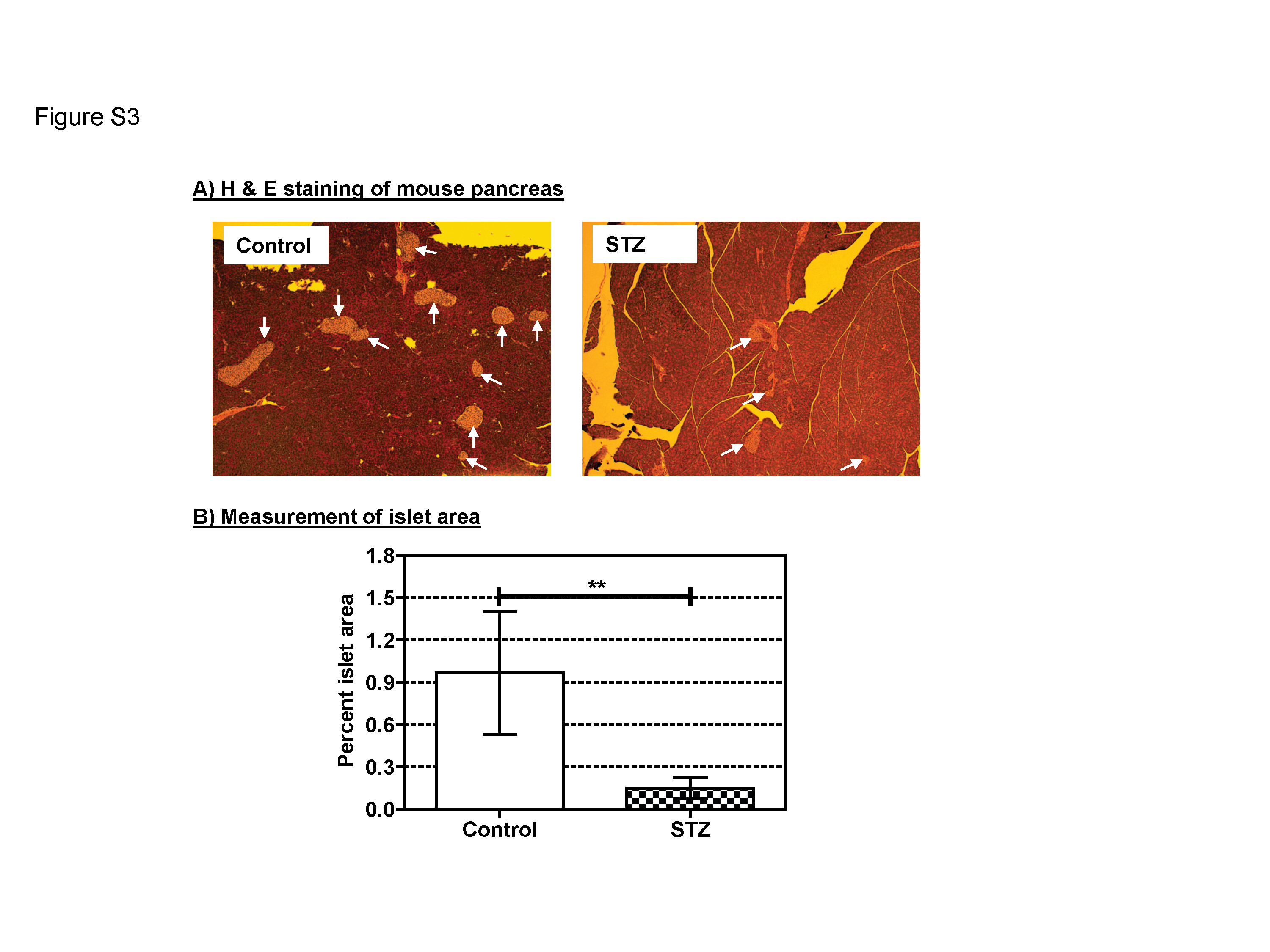

Supplement: Figure S3 — Significant reduction of islet area in STZ-treated diabetic mouse model. Pancreata removed from untreated control mice and STZ-treated mice that developed diabetes (n = 5 each) were fixed in 10% buffered formalin solution, embedded in paraffin and sectioned. The tissue sections were stained with hematoxylin and eosin (HE) using Tech-mate 1000 autostainer (Ventana, Tucson, AZ) by the City of Hope Anatomical Pathology Core. Stained sections were covered with Vectashield (Vector Laboratories, Burlingame, CA) and visualized using an Olympus IX51 fluorescent microscope equipped with an infinity 2 camera (Olympus America, Melville, NY). Pictures were captured using Infinity Analyze acquisition 5.0 software (Lumenera Corporation, Ottawa, Canada. A) Hematoxylin and eosin staining of pancreatic section of untreated control (left) and STZ-treated diabetic (right). Islets are indicated with arrows. B) The percent pancreatic islet area was quantified from histological sections of untreated control mice and STZ-treated mice using Fiji software (http://fiji.sc/wiki/index.php/Fiji). The data display the mean ± standard deviation (SD). The statistical significance was calculated with the Student t test for unpaired values and significance level indicated by asterisks (*** P<0.001). (TIFF) [file pone.0047942.s003.tiff]
